# Proteomic Investigation of *S*-Nitrosylated Proteins During NO-Induced Adventitious Rooting of Cucumber

**DOI:** 10.3390/ijms20215363

**Published:** 2019-10-28

**Authors:** Lijuan Niu, Jihua Yu, Weibiao Liao, Jianming Xie, Jian Yu, Jian Lv, Xuemei Xiao, Linli Hu, Yue Wu

**Affiliations:** College of Horticulture, Gansu Agricultural University, Lanzhou 730070, China; niulj0508@163.com (L.N.); liaowb@gsau.edu.cn (W.L.); xiejianming@gsau.edu.cn (J.X.); lvjian@gsau.edu.cn (J.L.); xiaoxm@gsau.edu.cn (X.X.); hull@gsau.edu.cn (L.H.); wuyue_gsau@163.com (Y.W.)

**Keywords:** Nitric oxide, *S*-nitrosylation, adventitious root development

## Abstract

Nitric oxide (NO) acts an essential signaling molecule that is involved in regulating various physiological and biochemical processes in plants. However, whether *S*-nitrosylation is a crucial molecular mechanism of NO is still largely unknown. In this study, 50 μM *S*-nitrosoglutathione (GSNO) treatment was found to have a maximum biological effect on promoting adventitious rooting in cucumber. Meanwhile, removal of endogenous NO significantly inhibited the development of adventitious roots implying that NO is responsible for promoting the process of adventitious rooting. Moreover, application of GSNO resulted in an increase of intracellular *S*-nitrosothiol (SNO) levels and endogenous NO production, while decreasing the *S*-nitrosoglutathione reductase (GSNOR) activity during adventitious rooting, implicating that *S*-nitrosylation might be involved in NO-induced adventitious rooting in cucumber. Furthermore, the identification of *S*-nitrosylated proteins was performed utilizing the liquid chromatography/mass spectrometry/mass spectrometry (LC-MS/MS) and biotin-switch technique during the development of adventitious rooting. Among these proteins, the activities and *S*-nitrosylated level of glyceraldehyde-3-phosphate dehydrogenase (GAPDH), tubulin alpha chain (TUA), and glutathione reductase (GR) were further analyzed as NO direct targets. Our results indicated that NO might enhance the *S*-nitrosylation level of GAPDH and GR, and was found to subsequently reduce these activities and transcriptional levels. Conversely, *S*-nitrosylation of TUA increased the expression level of *TUA*. The results implied that *S*-nitrosylation of key proteins seems to regulate various pathways through differential *S*-nitrosylation during adventitious rooting. Collectively, these results suggest that *S*-nitrosylation could be involved in NO-induced adventitious rooting, and they also provide fundamental evidence for the molecular mechanism of NO signaling during adventitious rooting in cucumber explants.

## 1. Introduction

Free radical nitric oxide (NO) is generated via non-enzymatic [1] and enzymatic pathways [2,3] in plants. As a multifunctional physiological regulator, NO has been shown to be involved in every aspect of plant growth and every developmental process in plants [4]. Furthermore, an increasing body of evidence has indicated that NO could play an essential role in response to various abiotic stresses [5,6,7].

Previous studies suggested that NO could exert its effects depending on the cyclic guanosine monophosphate (cGMP) signaling pathway [8,9]. For example, NO could promote the adventitious rooting of marigold through the cGMP-dependent pathway [10].

Additionally, the emerging picture is that NO also could operate biological functions through protein *S*-nitrosylation which is a NO-dependent posttranslational modification (PTM) [11,12]. It has been shown that NO groups could be covalently bound to cysteine (Cys) residues of target proteins, resulting in the formation of *S*-nitrosothiols during *S*-nitrosylation [11,13]. At present, increasing evidence demonstrates that *S*-nitrosylation might be involved in processes for regulating the growth, development, and stress responses in plants [14,15,16].

In general, proteomics deals with the large-scale determination of gene and cellular function directly at the protein level [17]. Recently, global protein *S*-nitrosylation has been identified using the proteomic approach. According to Hu et al. [18], more than 2200 *S*-nitrosylated proteins have been identified in mammals and plants. In the present work, several *S*-nitrosylated proteins have been identified using proteomic analyses in different plants. For example, Lindermayr et al. [19] identified proteins, which, when treated with NO, were involved in various pathways such as cytoskeleton organization, metabolic processes, redox homeostasis, as well as cellular signaling transduction. Moreover, Morisse et al. [20] identified 492 *S*-nitrosylated proteins and 392 sites in *chlamydomonas reinhardtii* cells, which were treated with *S*-nitrosoglutathione (GSNO). Moreover, 926 proteins that undergo nitrosylation have been identified in *Arabidopsis* [18]. Certain NO target proteins have been pointed out as important for regulating physiological and pathological cellular processes through proteomic and transcriptomic analyses [21,22,23]. Although the identification research on *S*-nitrosylation is increasing, the mechanism of *S*-nitrosylation during root development remains unclear. The aim of this study was to identify possible candidates for *S*-nitrosylation during adventitious rooting to reveal the biological function of NO at the protein level in plants. Therefore, we conducted this experiment to detect and identify the *S*-nitrosylated proteins during NO-induced adventitious rooting in cucumber explants. The objective of this study was to decipher the novel role of protein *S*-nitrosylation in the process of adventitious root development in order to further improve our understanding of NO signaling transduction in molecular mechanisms.

## 2. Results

### 2.1. Effect of Exogenous S-Nitrosoglutathione (GSNO) on Adventitious Rooting in Cucumber

**Figure 1:**
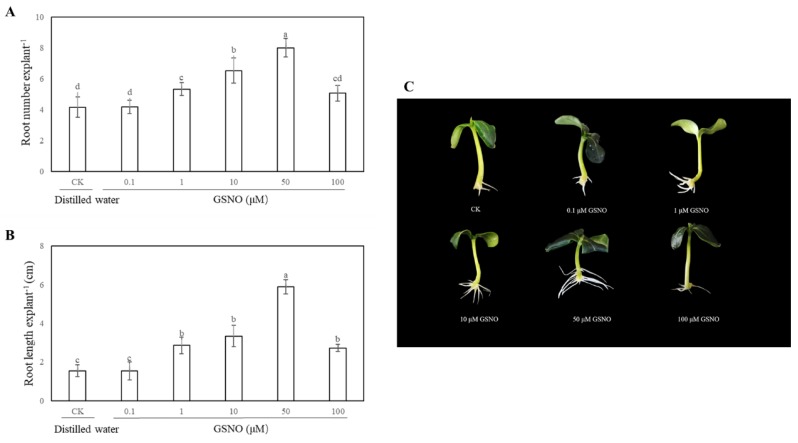
Effect of different concentrations of *S*-nitrosoglutathione (GSNO) on adventitious root development in cucumber explants. The primary roots were removed from hypocotyl of 5-day-old seedlings. Explants were incubated for 5 days with different concentrations of GSNO. The numbers (**A**) and root length (**B**) of adventitious roots were expressed as mean ± SE (*n* = 3). Ten explants were used per replicate. Photographs (**C**) were taken after five days of the treatments indicated. Bars with different lowercase letters were significantly different by Duncan’s multiple range test (*p* < 0.05). Bars with different lowercase letters were significantly different by Duncan’s multiple range test.

In order to access the effects of exogenous GSNO on adventitious root development of cucumber, explants were cultivated with different concentrations of GSNO (0, 0.1, 1, 10, 50 and 100 μM). As shown in Figure 1, there is no significant difference between the control and 0.1 μM GSNO. Meanwhile, lower concentrations of GSNO (1, 10, and 50 μM) significantly promoted the development of adventitious root. However, a higher dose of GSNO (100 μM) obviously decreased the number and length of adventitious roots, indicating exogenous GSNO could have a concentration-dependent effect on adventitious rooting. Moreover, the root number and length of 50 μM GSNO-treated explants increased by 92% and 280.60%, respectively, when compared with the control (Figure 1). These results revealed that 50 μM GSNO had the maximum biological effect on adventitious rooting. Therefore, 50 μM GSNO was used for the following experiments.

### 2.2. Effect of Nitric Oxide (NO) Scavenger on Adventitious Rooting in Cucumber

**Figure 2:**
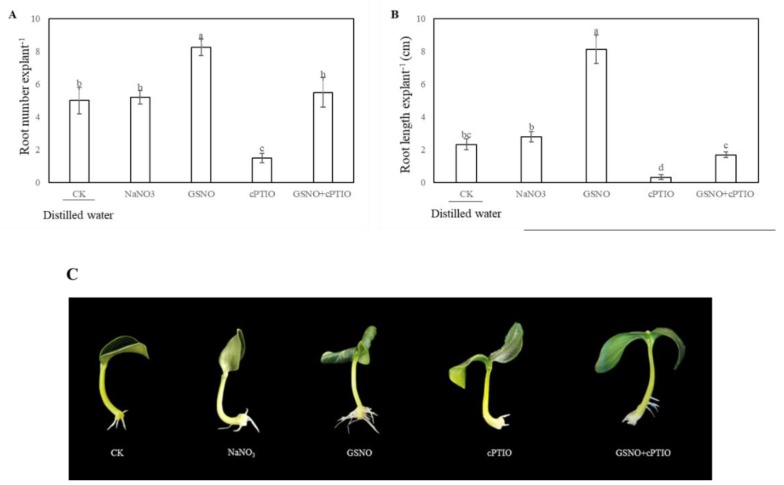
Effect of NO scavenger 2-(4-carboxy-2-phenyl)-4, 4, 5, 5-tetramethylimidazoline-1-oxyl-3-oxide (cPTIO) on adventitious root development in cucumber explants. The primary roots were removed from 5-day-old seedlings. Explants were then incubated for 5 days with distilled water (CK) or 100 μM sodium nitrate (NaNO_3_), 50 μM GSNO, 200 μM cPTIO, or 50 μM GSNO + 200 μM cPTIO. The numbers (**A**) and root length (**B**) of adventitious roots were expressed as mean ± SE (*n* = 3). Ten explants were used per replicate. Photographs (**C**) were taken after five days of the treatments indicated. Bars with different lowercase letters were significantly different by Duncan’s multiple range test (*p* < 0.05). Bars with different lowercase letters were significantly different by Duncan’s multiple range test.

In order to further confirm the effect of NO on adventitious rooting, NO scavenger 2-(4-carboxy-2-phenyl)-4, 4, 5, 5-tetramethylimidazoline-1-oxyl-3-oxide (cPTIO), and a normal product of NO decomposition, NaNO_3_ were applied in our research. Figure 2 showed that application of cPTIO alone clearly inhibited the adventitious root development. NaNO_3_ treatment as a control for NO decomposition had no effect on adventitious root development. However, GSNO + cPTIO treatment significantly reversed the inhibitive effect of NO scavengers (Figure 2). These results indicate that NO is responsible for the development of adventitious root in cucumber explants.

### 2.3. Effect of GSNO on the Levels of Total S-Nitrosothiol (SNO), and S-Nitrosoglutathione Reductase (GSNOR) Activity and Endogenous NO Level During the Development of Adventitious Roots in Cucumber

**Figure 3:**
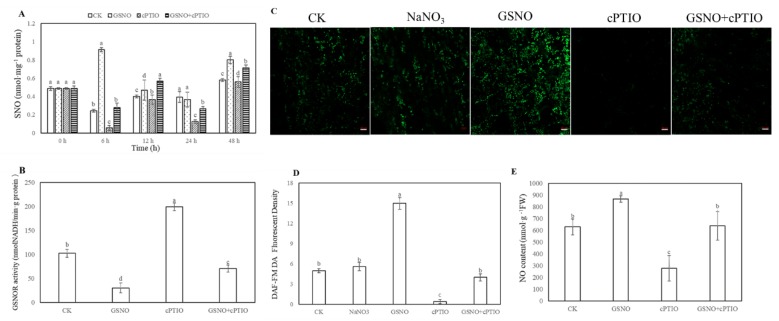
Effect of GSNO on the levels of total *S*-nitrosothiol (SNO) (**A**), *S*-nitrosoglutathione reductase (GSNOR) activity (**B**) and endogenous NO level (**C, D, E**) during the development of adventitious roots in cucumber. Explants were incubated with distilled water (CK) or 100 μM NaNO_3_, 200 μM cPTIO, 50 μM GSNO, or 50 μM GSNO + 200 μM cPTIO. The levels of total SNO (**A**) were determined during adventitious rooting. GSNOR activity (**B**) and endogenous NO levels (**C, E**) in cucumbers were detected after 6 h of treatment. 4-amino-5-methylamino-2′,7′- di aminofluoresceindiacetate (DAF-FM DA) was utilized to detect endogenous NO of a longitudinal section from the tip of the hypocotyls. Changes in fluorescence intensity of NO (**C**) were monitored by fluorescence microscopy after 6 h. The DAF-FM DA fluorescence density of endogenous NO (**D**) was analyzed by ImageJ software. Bars with different lowercase letters were significantly different by Duncan’s multiple range test (*p* < 0.05). Bars with different lowercase letters were significantly different by Duncan’s multiple range test.

To further elucidate whether *S*-nitrosylation was involved in the process of adventitious rooting, the level of endogenous *S*-nitrosothiol (SNO) was tested during adventitious rooting (Figure 3A). As shown in Figure 3A, during adventitious rooting, treatment with GSNO strikingly elevated the endogenous SNO level. At 6 h, nitroso groups with GSNO treatment reached the maximum value and were significantly higher than that of cPTIO treatment. On the contrary, lower *S*-nitrosoglutathione reductase (GSNOR) activity was found in GSNO treatment relative to that of control or cPTIO treatment at 6 h (Figure 3B). Additionally, application of GSNO treatment significantly enhanced the fluorescent intensity of NO production in cucumber hypocotyl. Meanwhile, there was no significant difference between distilled water (CK) treatment and sodium nitrate (NaNO_3_) treatment (Figure 3C,D). However, the production of endogenous NO was remarkably reduced in hypocotyl after NO scavenger treatment (Figure 3).

### 2.4. Identification of S-Nitrosylated Proteins During NO-Induced Adventitious Rooting in Cucumber

**Figure 4:**
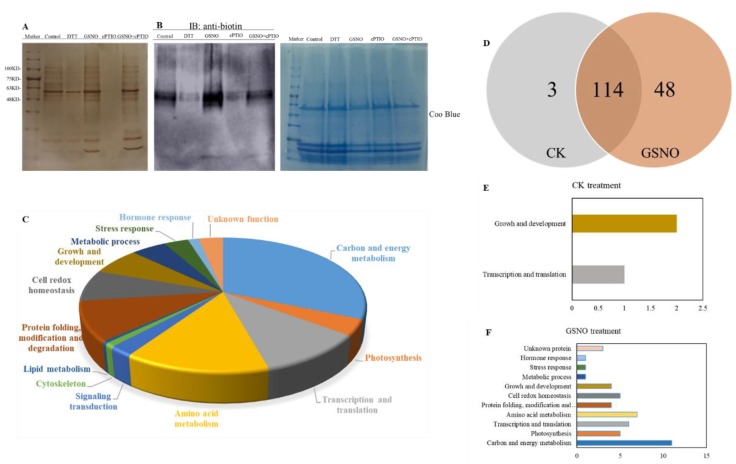
Identification of *S*-nitrosylated proteins during the development of adventitious rooting. Total *S*-nitrosylated proteins were detected through liquid chromatography/mass spectrometry/mass spectrometry (LC-MS/MS) and the western blotting method after explants were incubated with distilled water (CK) or 50 μM GSNO, 200 μM cPTIO, or 50 μM GSNO + 200 μM cPTIO for 6 h (**A, B**). Functional categorization of *S*-nitrosylated proteins from CK, GSNO and GSNO + cPTIO treatment (**C**). The number of *S*-nitrosylated proteins in CK and GSNO treated explants (**D**). Functional categorization of *S*-nitrosylated proteins from CK treatment alone (**E**). Functional categorization of *S*-nitrosylated proteins from GSNO treatment alone (**F**).

In order to further identify whether there exist possible candidates for *S*-nitrosylation during NO-induced adventitious rooting in cucumber explants, biotin switch detection and liquid chromatography/mass spectrometry/mass spectrometry (LC-MS/MS) were performed (Figure 4). As shown in Figure 4A,B, GSNO treatment obviously increased nitrosylation of proteins during adventitious rooting of cucumber, when compared to those of the control treatment. However, cPTIO treatment remarkably inhibited potential candidates for *S*-nitrosylation. Moreover, our results indicated that 167 proteins were identified from control, GSNO treatment, and GSNO + cPTIO treatment (Table 1). These identified proteins might be involved in various processes during adventitious rooting such as carbon and energy metabolism, photosynthesis, transcription and translation, and so on (Figure 4C). During adventitious rooting, approximately 40% were found to function in carbon and energy metabolism, 25.5% in the process of genetic information, and 8.5% in the growth and development process (Figure 4C). Additionally, identified proteins were found to function related to redox homeostasis, signaling transduction, and hormone response, about 9.7%, 3.0%, and 1.8%, respectively (Figure 4C). Among these proteins, three and 48 proteins were identified from the control and GSNO treatment, respectively (Figure 4D–F). As shown in Figure 4D, 114 proteins are common to both the control and GSNO treatment. These results implied that *S*-nitrosylation might be involved in NO-induced adventitious rooting in cucumber.

### 2.5. Effect of GSNO on the Activities and S-Nitrosylation Level of Tubulin Alpha Chain (TUA), Glutathione Reductase (GR), and Glyceraldehyde-3-Phosphate Dehydrogenase (GAPDH) During Adventitious Rooting

**Figure 5:**
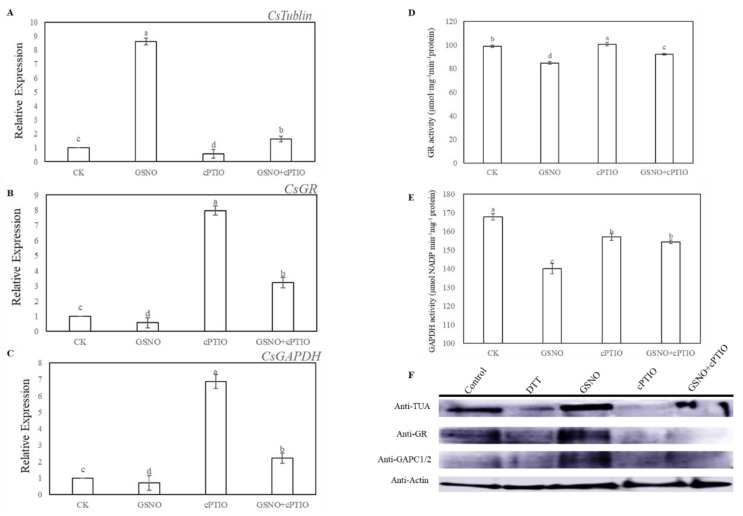
Effect of GSNO on the expression levels, enzymatic activities and *S*-nitrosylation level of tubulin alpha chain (TUA), glutathione reductase (GR), and glyceraldehyde-3-phosphate dehydrogenase (GAPDH) during adventitious rooting. Explants were incubated with distilled water (CK) or 200 μM cPTIO, 50 μM GSNO, or 50 μM GSNO + 200 μM cPTIO. *TUA*, *GR* and *GAPDH* expression level (**A, B, C**), and GR and GADPH activity (**D, E**) in cucumber explants was determined at 6 h of treatment. Immunoblot analysis of *S*-nitrosylated proteins in vivo (**F**). After biotinylation, proteins were purified with neutravidin-agarose, separated by sodium dodecyl sulfate-polyacrylamide gel (SDS-PAGE), and immunoblotted with anti-TUA, anti-GR, and anti-GAPDH antibodies. Bars with different lowercase letters were significantly different by Duncan’s multiple range test (p < 0.05). Bars with different lowercase letters were significantly different by Duncan’s multiple range test.

Here, tubulin alpha chain (TUA), glutathione reductase (GR), and glyceraldehyde-3-phosphate dehydrogenase (GAPDH) were selected as candidate proteins to further assess the level of nitrosylation during NO-induced adventitious rooting in cucumber. At 6 h, GSNO treatment significantly increased the expression level of *TUA*, and remarkably decreased the expression level and activities of GR and GAPDH (Figure 5A–E). However, exogenous application of GSNO significantly enhanced the nitrosylation level of these proteins, which was detected by the biotin switch technique (Figure 5D). On the contrary, the *S*-nitrosylation level of these proteins was largely blocked by the treatment of cPTIO (Figure 5D). Interestingly, removal of endogenous NO significantly inhibited the expression level of *TUA* but improved the expression level and activities of GR and GAPDH during the development of adventitious roots in cucumber (Figure 5A–E).

## 3. Discussion

In our study, the data presented herein demonstrated the evidence that there is a molecular mechanism of NO function to induce the development of adventitious rooting in cucumber. As previously reported in other researches, NO might play a critical role in affecting the root development [24,25,26]. For example, Yuan et al. [27] found the level of endogenous NO might be enhanced under cadmium (Cd) stress to inhibit the growth of root meristem in *Arabidopsis* through regulating auxin accumulation and transport. Alternatively, NO might act as a necessary factor affecting adventitious rooting [28,29,30]. According to our results, NO was indispensable for promoting adventitious rooting in cucumber (Figure 1 and Figure 2). Interestingly, research suggested that NO could partly exert its influence on the process of root growth and development through *S*-nitrosylation [31,32,33]. To investigate potential NO regulation of physiological processes through modifying cysteine residues of proteins [19], the changes of *S*-nitrosylation level and endogenous NO content during adventitious rooting of cucumber explants were analyzed (Figure 3). Application of exogenous NO significantly increased the level of endogenous SNO and endogenous NO production. However, SNO and NO level with cPTIO treatment significantly were lower than those of the control and GSNO treatment, implying that NO might enhance the endogenous nitrosylation level during adventitious rooting (Figure 3A,C–E). Previously, Wang et al. [32] found that SNP could enhance the level of SNO. Our results indicated that NO might affect process of adventitious rooting through enhancing the endogenous nitrosylation. Moreover, it is known that GSNOR can regulate global levels of *S*-nitrosylation [34,35] Additionally, Lin et al. [36] found that *S*-nitrosoglutathione reductase (*OsGSNOR)* overexpression transgenic plants were detected with a lower SNO content indicating that GSNOR might play a vital role in SNO homeostasis. As mentioned above, NO might inhibit the activity of GSNOR1 preventing *S*-nitrosoglutathione scavenging [35]. As depicted in Figure 3B, a lower GSNOR activity was detected in GSNO treatment, which also suggested that GSNOR regulates the total level of SNOs during NO-induced adventitious rooting in cucumber.

For a deeper insight, *S*-nitrosylated proteins were identified during adventitious rooting of cucumber (Figure 4; Table 1). Among these proteins, a large amount of the *S*-nitrosylated proteins identified were closely related to carbon and energy metabolism, implying this process could be regulated by *S*-nitrosylation, during adventitious rooting of cucumber. Previous research suggested that carbohydrates and nitrogen compounds might provide nutrition and energy during adventitious root formation and development [37]. In our study, for example, pyruvate kinase, malate dehydrogenase, and malate synthase were involved in the tricarboxylic acid (TCA) cycle, which acts as an iconic process for carbohydrate metabolism [38]. However, the molecular mechanisms of these protein functions during the development of adventitious roots are still not established. Here, our results imply that these proteins may be *S*-nitrosylated during NO-induced adventitious rooting of cucumber. Moreover, cytoskeleton change might affect cell shape and translocate organelles which could enhance cell response to intracellular and extracellular signaling [39]. Potential candidates of *S*-nitrosylation during adventitious rooting in cucumber are also related to cytoskeleton structure including tubulin α and tubulin β [40]. Tubulin α and tubulin β have been demonstrated to be *S*-nitrosylated in mammals and plants [40,41]. These results indicate that the *S*-nitrosylation of tubulin variants could act as an important mediator in NO-promoted development of adventitious roots in cucumber. Additionally, another cluster of potential candidates for *S*-nitrosylation includes metabolic enzymes such as GAPDH, glucose-6-phosphate isomerase, fructose-bisphosphate aldolase, phosphoglycerate kinase, and so on (Table 1). Previous studies have reported that H_2_O_2_ treatment might affect fructose-1,6-biphosphate aldolase and 2-phosphoglycerate hydrolase undergoing *S*-glutathionylation [42]. Meanwhile, Lindermayr et al. [19] suggested that the glycolysis-related enzymes are sensitive to *S*-nitrosylation. Thus, these metabolism enzymes, which are identified as targets for *S*-nitrosylation, imply that *S*-nitrosylation of metabolic proteins could mediate adventitious root development.

In our study, there were 116 *S*-nitrosylated proteins from both control and GSNO treatments (Figure 4C). These proteins participated in different processes of cellular metabolism, such as lipid metabolism, transcription and translation, hormone response, and signaling transduction (Figure 4D,E). As a consequence, these *S*-nitrosylated proteins with different functions might play a vital role in affecting the process of adventitious rooting. As previously reported in Wang et al. [32], NO could inhibit the growth of primary roots through *S*-nitrosylation of plastidial GAPDH. Our results indicated that NO could enhance the *S*-nitrosylation level of GAPDH, however, it was shown to decrease the expression level and activity of GAPDH during adventitious rooting (Figure 5C,E). In animals, some research has demonstrated that NO could inhibit GAPDH activity through *S*-nitrosylation [43,44]. Additionally, GAPDH activity was clearly inhibited by exogenous NO during NO-repression of the process of primary root growth [24]. These results might indicate that GAPDH is a key target for NO-specific PTM. Furthermore, we demonstrated evidence for the first time that GR and TUA could be over-nitrosylated under NO treatment during adventitious rooting (Figure 5). According to a previous study, GR had been shown to play an essential role for cell redox homeostasis [45]. Moreover, TUA has been found to play an essential role in cytoskeleton development [46]. The development of adventitious roots may be closely related with cell division and cell growth [47]. As depicted in Figure 5A, NO significantly increased the expression level of *TUA*, suggesting that the cell cycle process plays a vital role during adventitious root growth [48]. In addition, Begara-Morales [49] found that chloroplastic and cytosolic GR in peas are *S*-nitrosylated by GSNO, however, NO-based modification did not significantly affect this protein activity. In a previous study on mammal cells, an inhibitory effect on GR activity was shown after exposure to GSNO for a longer time [50]. According to our results, GSNO significantly decreased the expression level and activity of GR during adventitious rooting, implying that *S*-nitrosylation of GR induced by GSNO might inhibit protein activity and this change could be related to the development of adventitious roots in cucumber. Although some *S*-nitrosylation of proteins during adventitious rooting have been identified, whether the activities and functions of these identified proteins have been changed due to *S*-nitrosylation directly, needs to be further investigated. In the future, these results could provide valuable information for future investigations.

## 4. Materials and Methods

### 4.1. Plant Materials

Cucumber (*Cucumis sativus* ‘BaiLv 1′) seeds were supplied by the Gansu Academy of Agricultural Sciences, Lanzhou, China. The seeds were germinated in petri dishes on filter papers soaked with distilled water and maintained at 25 ± 1 °C for 6 days with a 14 h photoperiod (photosynthetically active radiation = 200 μmol s^–1^ m^–2^). After removing the primary roots of 6-day-old seedlings, the cucumber explants were then maintained under the same conditions of temperature and photoperiod for another 5 days under different treatments as indicated below. These media were changed every day in order to keep the solution fresh. The number and length of adventitious roots per explant were counted and measured.

### 4.2. Treatments of Explants

Explants were placed in petri dishes containing distilled water (control) and different concentrations of *S*-nitrosoglutathione (GSNO, a donor of NO, Sigma, St Louis, MO, USA) as indicated in Figure 1 and kept at 25 ± 1 °C 200 μM 2-(4-carboxy-2-phenyl)-4, 4, 5, 5-tetramethylimidazoline-1-oxyl-3-oxide (c-PTIO; Sigma, St Louis, MO, USA), 100 μM sodium nitrate (NaNO_3_, degradation product of NO, Solarbio, Beijing, China) was added alone and with a suitable concentration of GSNO. The concentrations of NO scavenger and NaNO_3_ were based on the results of a preliminary experiment.

### 4.3. Determination of Endogenous SNO Content, NO Production, and GSNOR Activity

SNO content was determined as described by Feechan et al. [34] with minor modifications. Fresh cucumber explants were homogenized with extraction buffer (50 mM Tris-HCl, pH 8.0), 150 mM NaCl, and 1 mM protease inhibitor phenylmethanesulfonyl fluoride (PMSF) in an ice bath for 20 min. The centrifugation was performed at 10,000 rpm for 15 min at 4 °C. The absorbance of the mixture reaction, which includes 1.5 mL of the supernatant, 1.5 mL of 0.1% N-(1-naphthyl)-ethylenediamine, and 1% sulfanilamide, with and without adding HgCl_2_, was taken for 20 min in the dark. SNO content was recorded photometrically at 540 nm [34].

The level of endogenous NO in cucumber hypocotyls at 6 h was detected by NO fluorescent probe 4-amino-5-methylamino-2′,7′- diaminofluoresceindiacetate (DAF-FM DA) [51]. The hypocotyls were loaded with 5 μM DAF-FM DA for 30 min at 37 °C in the dark. The samples were then washed three times with fresh buffer. DAF-FM DA fluorescence was visualized using a laser scanning confocal microscope (Leica TCS SL; Leica Microsystems, Wetzlar, Hessen, Germany). The excitation wavelength was 488 nm and the emission wavelength was 515 nm.

NO content was measured according to the Greiss reagent method with some modifications [52]. A quantity of 0.2 g of explants was finely frozen in liquid nitrogen with the extract mixture (4 mL of 50 mM ice cold acetic acid buffer, containing 4% zinc diacetate). After that, centrifugation was performed at 10,000× *g* for 15 min at 4 °C, and then the supernatants were collected. For each sample, 0.1 g of charcoal was added. After that, the supernatants were filtered and collected again, and then 1 mL of the mixture was pipetted into 1 mL of Greiss reagent. They were allowed to react for 30 min at room temperature. Then the absorbance was assayed at 540 nm.

*S*-nitrosoglutathione reductase (GSNOR) activity was measured using the method of Durner et al. [53]. Samples were homogenized with 20 mM Tris-HCl (pH 8.0, 0.2 mM NADH, and 0.5 mM EDTA) and centrifuged at 10,000 rpm for 20 min at 4 °C. The reaction was started by adding GSNO and the absorbance of the sample was measured at 340 nm.

### 4.4. Biotin-Switch Assay and Identification of Biotinylated Proteins

Cucumber explants were ground in liquid nitrogen, extracted by HEN-2 Buffer (250 mM Hepes-NaOH, EDTA, neocuproine and proteinase inhibitor), followed by centrifugation at 13,000 *g* for 10 min at 4 °C. Then, extracted protein was incubated in blocking buffer (250 mM Hepes, EDTA, SDS, methylmethane thiosulphonate (MMTS)) for 30 min at 50 °C under dark conditions. Subsequently, the MMTS was removed by cold acetone. The protein was resuspended with HEN-1 buffer (250 mM Hepes, EDTA, SDS) and 1 mM sodium ascorbate and biotin-HPDP (Sigma, St Louis, MO, USA) were added for labeling. The *S*-nitrosylated proteins were identified by LC-MS/MS and measured by immunoblot analysis [54].

### 4.5. Western Blotting

For western blot analysis, proteins from different treatments were resolved using SDS-PAGE on 12% polyacrylamide gels, and transferred to polyvinylidene difluoride membranes (PVDF, Novex, San Diego, CA, USA) utilizing a wet transfer device (BioRad, Barcelona, Spain) at 105 V for 70 min at 4 °C. The immunoreaction was performed with rabbit polyclonal antibodies against Biotin (1:2500) (Agrisera, Vännäs, Sweden), TUA (1:5000) (Agrisera, Vännäs, Sweden), GR (1:5000) (Agrisera, Vännäs, Sweden), and GAPDH (1:2000) (Agrisera, Vännäs, Sweden), Actin (1:2500) (Agrisera, Vännäs, Sweden). The blot was incubated in secondary antibody (goat anti-rabbit IgG), diluted to 1:10,000, for 1 h at 25 °C.

### 4.6. GR, GAPDH Activity

GR activity was determined according to Foyer et al. [55]. A 0.2 g quantity of explant was ground in liquid nitrogen with the extract mixture, followed by centrifugation at 12,000 *g* for 20 min at 4 °C. Then, a total of 100 μL of enzyme extract was transferred into 2 mL of reaction mixture (25 mM sodium phosphate buffer, pH 7.0, 0.1 mM EDTA, 0.5 mM oxidized glutathione (GSSG), 0.12 mM NADPH). GR activity was evaluated by measuring the decrease in absorbance at 340 nm due to NADPH oxidation.

The measurement of GAPDH activity was according to the method of Piattoni et al. [56]. Crude protein extraction was performed with 200 μL reaction buffer (50 mM Tris-HCl, pH 8.5, 10 mM sodium arsenate, 2 mM NAD^+^, 1 U/mL aldolase, 1.2 mM fructose-1,6-diphosphate) at 30 °C. Then, the reaction was monitored at 340 nm.

### 4.7. Gene Expression Analyses by RT-qPCR

The method of real time RT-PCT (RT-qPCR) analyses and statistical data analyses reference the procedure of Zhao et al. [57]. The cDNA was amplified using the following primers: for Actin (accession No. AB010922.1), F: TTGAATCCCAAGGCGAATAG and R: TGCGACCACTGGCATAAAG; for *CsTUA* (accession No. AJ715498.1), F: 5′-TTGTTCCTGGAGGCGATCTT-3′ and R: 5′- ACAAATGCGCGCTTAGCATA-3′. For *CsGR* (accession No. NM_001308836.1): F: 5′- GATATGAGAGCCGTGGTTGC-3′ and 5′- AGTCGCAAACAACAC AGCAT-3′; for CsGAPDH (accession No. NM_001305758.1), 5′- TGACGA GTCCATCATCAGCAATGC-3′ and 5′- CAATGTTGAGTGCAGCAGCTCTTG-3′. The expression analyses were conducted three times independently.

### 4.8. Statistical Analysis

The statistical analyses was analyzed using the Statistical Package for Social Sciences for Windows (version 13.00; SPSS, Inc., Chicago, IC, United States) and statistical differences were analyzed through Duncan’s multiple range test (*p* < 0.05). In the analysis of variance (ANOVA), results were expressed as the mean values ± SE from three independent replicates.

## 5. Conclusion

**Figure 6:**
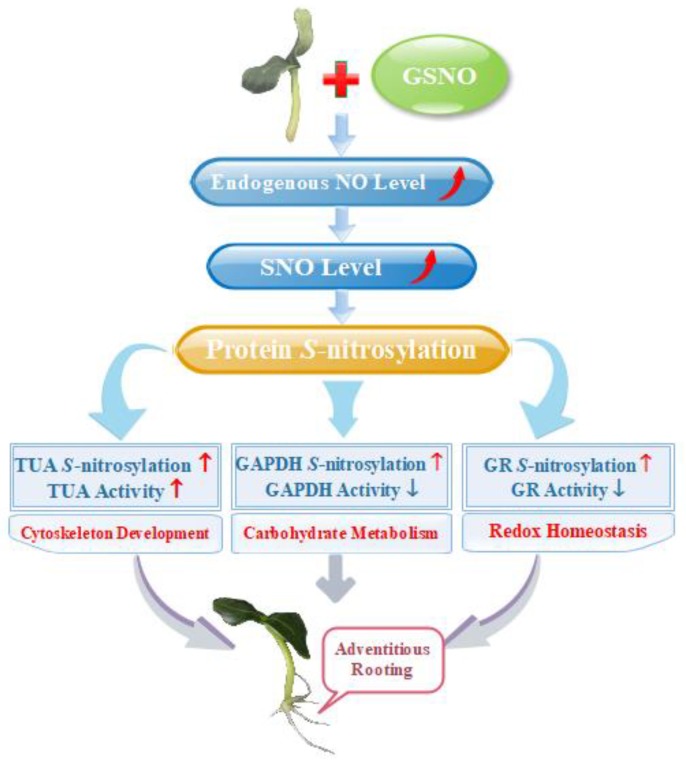
Schematic model of NO-induced *S*-nitrosylation during adventitious rooting in cucumber. NO-enhanced endogenous NO concentration and SNO levels, which triggers *S*-nitrosylation of proteins to induce adventitious root development. Differential *S*-nitrosylation of TUA, GAPDH and GR might regulate various pathways during NO-promoted the development of adventitious roots. The increase is indicated by the red arrow. The decrease is indicated by the blue arrow.

Taken together, the evidence presented in this study showed that there are a series of *S*-nitrosylated proteins during NO-induction of the development of adventitious roots, which highlights the effect of NO-based posttranslational modification on regulating the development of adventitious roots in cucumber. Moreover, differential *S*-nitrosylation of key proteins regulated various pathways during adventitious rooting (Figure 6). Thus, our work demonstrated that *S*-nitrosylation process is an essential modulator during adventitious rooting of cucumber. Further work should focus on deciphering the function of such *S*-nitrosylated proteins on affecting adventitious root development. Therefore, corresponding genetic and proteomic evidences should be provided to further investigate mechanisms.

## Figures and Tables

**Table 1:** *S*-nitrosylated proteins identified from the control, S-nitrosoglutathione (GSNO), and GSNO + 2-(4-carboxy-2-phenyl)-4, 4, 5, 5-tetramethylimidazoline-1-oxyl-3-oxide (cPTIO) treatment during adventitious rooting of cucumber seedlings.

Accession Number	Protein Name	Mol Mass	Peptide Sequence
A0A0A0K9P5	11S globulin subunit beta-like	54 kDa	SSLLAFLC^11^LAVFING NGFEETVC^299^TLRLKHN
A0A0A0K674	26S protease regulatory subunit 7	47 kDa	AKKVNDLC^56^GIKESDT QPLQVARC^91^TKIINPN MARSKKAC^263^IVFFDEV
A0A0A0K3C4	26S proteasome non-ATPase regulatory subunit 2 homolog	98 kDa	GLIYLGSC^539^NEEVAQA
A0A0A0LQ32	4-alpha-glucanotransferase	64 kDa	YSGQDANC^140^GNTLLIS
A0A0A0KAJ9	60S ribosomal protein L3	44 kDa	KDDATKPC^41^RLTAFLG
A0A0A0KXM8	6-phosphogluconate dehydrogenase, decarboxylating	53 kDa	AYLEKGDC^103^IIDGGNE
Q08375	Acetyl-CoA acyltransferase (3-ketoacyl-coa thiolase)	48 kDa	SIENAQNC^191^LLPMGVT FASQFVYC^370^RNKLGLD LGATGARC^401^VATLLHE AVFERGDC^440^VDELCNA
A0A0A0LFR2	Acetyltransferase component of pyruvate dehydrogenase complex	58 kDa	NRSQFLQC^75^QRGVSMM YYLTVDTC^341^VDKLMDL FMSVTLSC^509^DHRVIDG
A0A0A0KHD6	Aconitate hydratase	95 kDa	PAVVDLAC^103^MRDAMNR ALVAKKAC^442^ELGLEVK
A0A0A0KJ21	Actin	41 kDa	EDIQPLVC^12^DNGTGMV TYNSIMKC^287^DVDIRKD
A0A0A0KRC5	Acyl-coenzyme A oxidase	73 kDa	QHLMESTC^457^KVQKAED SARMSVEC^486^AKRLSQF KDQLQKLC^544^SIYALFT
A0A0A0LNE3	Adenosylhomocysteinase	53 kDa	EMPGLMAC^42^RTEFGPS
A0A0A0KSC6	Adenylosuccinate lyase	60 kDa	MEIGANC^7^RVLDQPR LEFFHFSC^186^TSEDINN
A0A0A0K9F9	Aldehyde dehydrogenase family 7 member B4	54 kDa	QYMRRSTC^490^TINYGNE
H6WX41	Alkaline alpha galactosidase 3	86 kDa	HHTDAVYC^441^AKQTAVV SSAKPRQC^744^IVDSSVV
A0A0A0KMH9	Alpha-mannosidase	114 kDa	MEKQANSC^8^LPFSFLV NNSIQGAC^76^VQNVLDS QPKILSQC^470^PLLNISF
A0A0A0L5C9	Aminopeptidase	99 kDa	QPSSIQAC^82^EVSQILV AFALSMAC^587^QQSVTSL
A0A0A0KF04	Aminotransferase 2	44 kDa	DHTIKAVC^142^IVHNETA
A0A0A0LEK8	Aspartate aminotransferase	50 kDa	NRVTTVQC^163^LSGTGSL
G3EIZ8	ATP synthase subunit alpha	54 kDa	AESETLYC^202^VYVAIGQ
A0A2D0UXD2	Betaine aldehyde dehydrogenase	54 kDa	AKLEAIDC^100^GKPLEEA
A0A0A0K2H5	Beta-xylosidase/alpha-L-arabinofuranosidase 2-like	84 kDa	LAGLDLDC^344^GDFLGKH PGCANVAC^485^TSAQLDE
A0A0A0KYI1	Biotin carboxylase	58 kDa	MDAAMPLC^8^KSARAPS KLADESVC^117^IGEAPSS SAAVSRGC^142^TMLHPGY
A0A0A0LD02	Carbonic anhydrase	35 kDa	STASINTC^9^LFSLNKS ACSDSRVC^167^PSHVLDF
G8EX76	Chloroplast transketolase	80 kDa	EGIANEAC^246^SLAGHWG
A0A0A0LCU8	Coatomer subunit beta	106 kDa	MEKSC^5^TLLVHFD STAVIYEC^262^AGTLVSL RAAANTYC^284^QLLLSQS MKSTNMKC^879^LTPISAL
A0A0A0LBW6	D-3-phosphoglycerate dehydrogenase	63 kDa	AAATEHGC^144^LVVNAPT
A0A0A0KG56	Dihydrolipoyl dehydrogenase 2, chloroplastic-like	59 kDa	KLVPHVYC^393^IGDANGK
A0A0A0LTJ3	Elongation factor Ts, mitochondrial	121 kDa	TGAGMMDC^693^KKALAES TGAGMMDC^936^KKALSET
A0A0A0K581	Eukaryotic translation initiation factor 3 subunit B	60 kDa	TTKTLGYC^112^FIEYGTP
A0A0A0LC36	Eukaryotic translation initiation factor 3 subunit C	106 kDa	TKARAMLC^519^DIYHHAL SWDQPSGC^785^IIFHDVT
A0A0A0L3P3	Ferredoxin--NADP reductase, chloroplastic	46 kDa	DSKTVSLC^213^VKRLVYT
A0A0A0K8H3	Fructose-1,6-bisphosphatase, cytosolic	36 kDa	LVSSGRTC^95^ILVSEED
A0A0A0KKE4	Fructose-bisphosphate aldolase	38 kDa	MSC^3^YRGKYAD
A0A0A0KEY8	Glucose-1-phosphate adenylyltransferase	57 kDa	PNLKRKLC^58^ISSLIAD
A0A0A0LRW2	Glucose-6-phosphate isomerase	67 kDa	MASISGIC^8^SSSPSLK AVLNEASC^559^KEPVEPL
A0A0A0KPY1	Glutamate decarboxylase	56 kDa	MVDENTIC^205^VAAILGS KKKTNGVC^499^
A0A0A0K488	Glutamate-1-semialdehyde 2,1-aminomutase 2, chloroplastic-like	54 kDa	SVGIGLPC^47^STKLSHT
A0A0A0K8Q7	Glutathione reductase	59 kDa	AGGVGGTC^122^VIRGCVP
A0A0A0K8C1	Glyceraldehyde-3-phosphate dehydrogenase	36 kDa	NIVSNASC^154^TTNCLAP NASCTTNC^158^LAPLAKV
A0A0A0LN17	Glycine cleavage system P protein	113 kDa	TFVISNNC^252^HPQTIDI NPASAAMC^688^GMKIVSV
A0A0A0LAN5	Glycosyltransferase	55 kDa	QLTPRPNC^123^IISDMCI
A0A0A0KHX5	Glyoxysomal fatty acid beta-oxidation multifunctional protein MFP-a	79 kDa	MC^2^HALLVTI NLKHTIAC^303^IDAVETG
A0A0A0LNA7	Guanosine nucleotide diphosphate dissociation inhibitor	49 kDa	SEGETAKC^278^KKVVCDP
A0A0A0K921	Heat shock 70 kDaa protein 15-like	92 kDa	VIDQLVYC^704^INSYREA
A0A0A0KXG3	Heat shock protein 70	70 kDa	NMDLFRKC^319^MEPVEKC CMEPVEKC^326^LRDAKMD MKELESIC^609^NPIIAKM
A0A0A0K5T7	Ketol-acid reductoisomerase	63 kDa	NISVIAVC^242^PKGMGPS CMDILYEC^394^YEDVASG
A0A0A0LXB9	L-ascorbate oxidase	65 kDa	YMFWSPDC^54^VENIVMG GTASISQC^116^AINPGET ELSGKEKC^236^APFILHV IPPKALAC^574^GSTALVK
A0A0A0L5B9	Lon protease homolog 2, peroxisomal	98 kDa	DLKLASAC^757^ESNLLEG
A0A0A0LR30	Lsocitrate lyase	64 kDa	QLKTFSEC^320^VTDAIMN
A0A0A0L0E4	Malate dehydrogenase	36 kDa	CTAIAKYC^142^PNALVNM
A0A0A0LUC5	Malate synthase	65 kDa	KGMYKEAC^533^KMFTRQC
A0A0A0L5H2	Methionine *S*-methyltransferase	120 kDa	VDSFLALC^15^QQSGDAA QLERIVGC^210^IPQILNP HALSVYSC^364^QLLQPNQ HLPAQREC^664^DKSASSR CGWDVIEC^997^HAGVSVV ADFKRIAC^1082^SS
A0A0A0LEZ3	Methionine synthase	84 kDa	IPSNTFSC^64^YDQVLDT HLVVSTSC^328^SLLHTAV
A0A0A0LIC6	Methylenetetrahydrofolate reductase	72 kDa	ETMMHLTC^128^TNMPVEK YEKFMKYC^446^LGKLRSS
A0A0A0KI79	Mg-protoporphyrin IX chelatase	45 kDa	KGRPQVQC^60^NVATEIN KVKISRVC^350^AELNVDG
A0A0A0LN97	Multicopper oxidase	60 kDa	DGVYGTTC^99^PIPPGKN
A0A0A0KIJ0	Ncharacterized protein	55 kDa	IEPVPESC^99^VSTLEER
A0A0A0L679	Phospho-2-dehydro-3-deoxyheptonate aldolase	57 kDa	FLLQGGDC^124^AESFKEF NSRYHTHC^479^DPRLNAS
A0A0A0KEF3	Phosphoglycerate kinase	50 kDa	QVVKADDC^177^IGPEVEK
A0A0A0KTJ4	Phospholipase D	92 kDa	YFSQRRGC^178^KVTLYQD KFYEPHRC^209^WEDVFDA LFPESIEC^736^VKSVNQL
A0A0A0L987	Phosphoribulokinase	46 kDa	****MAVC^4^TVYTTQS
A0A0A0L989	Polyadenylate-binding protein	71 kDa	AFGSILSC^146^KVALDSS
A0A0A0K809	Presequence protease 1, chloroplastic/mitochondrial-like	122 kDa	VFLRSLTC^12^SSLVCNR RGKAMSGC^743^AEDLFNL SLLSRKNC^847^LVNITAD
A0A0A0K8X9	Protease Do-like 2, chloroplastic	68 kDa	AAAMASSC^9^FSPFDST VLARGVDC^204^DIALLSV LKFGNLPC^230^LQDAVTV AAIAASSC^571^ILRDYGI
A0A0A0LRK5	Purple acid phosphatase	54 kDa	VLCDLGVC^26^NGGITSG
A0A0A0L0U0	Pyrophosphate--fructose 6-phosphate 1-phosphotransferase subunit alpha	67 kDa	ETFAEAKC^208^PTKVVGV ASHVALEC^276^TLQSHPN RTIVKPGC^584^SQEVLKA
A0A0A0KH95	Pyrophosphate--fructose 6-phosphate 1-phosphotransferase subunit beta	61 kDa	LKTRVIGC^224^PKTIDGD SFGFDTAC^247^RIYAEMI
A0A218KBQ1	Pyruvate kinase	55 kDa	KPGNNILC^143^SDGTITL QKMMIYKC^287^NLAGKPV AVLDGTDC^328^VMLSGES
A0A0A0KAU8	RuBisCO large subunit-binding protein subunit alpha	64 kDa	LSSASILC^14^SSHKSLR
A0A0A0KFZ8	RuvB-like helicase	51 kDa	PQTKFVQC^224^PDGELQK
A0A0A0KBZ1	*S*-(hydroxymethyl)glutathione dehydrogenase	40 kDa	TQGQVITC^10^KAAVAWE GVDYSFEC^271^IGNVNVM
C4PAW8	Sedoheptulose-1,7-bisphosphatase	42 kDa	GLIRLLTC^93^MGEALRT SHFCKYAC^148^SEEVPEL
A0A0A0K8A3	Selenium-binding protein 2-like	53 kDa	KDTGYVGC^277^ALTSNMV
A8CM21	Stachyose synthase	96 kDa	SSAINKGC^383^TSCSCKA GLTNMFNC^792^SGTIQHL
A0A0A0KGA1	Succinate-semialdehyde dehydrogenase	58 kDa	GPALASGC^230^TVVIKPS NSGQTC^346^VCANRILVQ
A0A0A0LVU2	T-complex protein 1 subunit delta	57 kDa	RSLHDALC^404^VVRCLVN AITLATEC^519^VRMILKI
A0A0A0LZU0	T-complex protein 1 subunit eta	60 kDa	FADRDIFC^313^AGRVAEE NAATEAAC^517^LILSVDE
A0A0A0LLK5	Tocopherol cyclase	57 kDa	PLCGIHHC^16^SFKLVEA
A0A0A0KBL8	Transketolase, chloroplastic	80 kDa	NRSSRSRC^65^GVVRASV EGIANEAC^249^SLAGHWG
A0A0A0K6A8	Tubulin alpha chain	49 kDa	GIQVGNAC^20^WELYCLE TIQFVDWC^347^PTGFKCG AKVQRAVC^376^MISNSTS
A0A0A0LCY8	Tubulin beta chain	50 kDa	LHIQGGQC^12^GNQIGAK ATMSGVTC^238^CLRFPGQ NNVKSTVC^354^DIPPTGL
A0A0A0K9N4	Ubiquitin carboxyl-terminal hydrolase 6	54 kDa	YMNSTLQC^121^LHSVPEL MQQDAEEC^200^WTQLLYT ESVYSLKC^256^HISQEVN
A0A0A0KZ30	UDP-glucose 6-dehydrogenase	52 kDa	MVKIC^5^CIGAGYV TKEAHAVC^417^ILTEWDE
A0A0A0KZU3	Gamma aminobutyrate transaminase 2	56 kDa	TNPKLGSC^18^AKDVAAL
A0A0A0LHR0	PALP domain-containing protein	58 kDa	SSPFTLVC^36^SSATSDS
A0A0A0LQL1	Uncharacterized protein	110 kDa	LARGQLRC^391^IGATTLE
A0A0A0LTW3	UVR domain-containing protein	96 kDa	RRRKASRC^26^VPRAMFE LARGELQC^343^IGATTLD
A0A0A0KSQ4	Probable nucleoredoxin 1	63 kDa	WICEGGVC^559^RKA
A0A0A0L5E7	Uncharacterized protein	43 kDa	QQFTGLRC^13^APLSSSR
A0A0A0LNR8	Peptidase_S9 domain-containing protein	85 kDa	ILSGEVSC^428^ISPANSN PVKDVSNC^514^LTKGASE AAARNPVC^653^NLALMVG
A0A0A0KN12	Oxalate--CoA ligase-like	55 kDa	KLRFIRS^291^CSASLAPS
A0A0A0K983	Uncharacterized protein	69 kDa	TTDGKTNC^422^LNAAVGT AMVTQAYC^569^DVPFSYT
A0A0A0KI31	Glyoxysomal fatty acid beta-oxidation Multifunctional protein MFP-a	79 kDa	GLEVAMAC^124^HARLSTK NLKHPLVC^251^IDVVETG
A0A0A0KIK3	enolase isoform	47 kDa	QIKTGAPC^408^RSERLAK
A0A0A0KL58	AA_TRNA_LIGASE_II domain-containing protein	51 kDa	TATERTLC^402^CILENYQ ATERTLC^403^CILENYQK
A0A0A0KPT0	Protein kinase domain-containing protein	127 kDa	RGAAKGLC^974^FLHHNCI YLEITLRC^1124^VEEFPSK
A0A0A0KQJ3	Alpha-amylase 3, chloroplastic isoform	101 kDa	LDPLLYHC^13^AKGKHRF RPCSFTYC^37^PNKLLCH NWELTVGC^112^NLAGKWI ISVSVRKC^292^SETTKYL
A0A0A0KTH8	Malate dehydrogenase, chloroplastic	48 kDa	SRTSRVTC^49^SINQVEA CNTNALIC^230^LKNAPKI ELLAEKRC^413^VAHLTGE
A0A0A0KW04	2-hydroxyacyl-CoA lyase	60 kDa	DISEIPNC^154^VARVLNS RSLAIGKC^274^DVALVVG
A0A0A0KWS0	2,3-bisphosphoglycerate-independent Phosphoglycerate mutase	61 kDa	NGVRTFAC^356^SETVKFG
A0A0A0L7Y5	11S globulin seed storage protein 2-like	57 kDa	SSGLIVKC^260^DEEMSFL NGIEETVC^297^TARVQHN
A0A0A0LFS9	Cell division control protein 48 homolog E	89 kDa	CTEAALQC^426^IREKMDV KARQSAPC^576^VLFFDEL
A0A0A0LHX3	Uncharacterized protein	71 kDa	NTPQQLAC^176^IDVIEDG KVPLCIPC^201^EDKVFRE
A0A0A0LJ13	Triosephosphate isomerase, chloroplastic	32 kDa	EGLGVIAC^177^IGELLEE
A0A0A0LTR4	Beta-glucosidase 44-like	57 kDa	LPVVCMLC^14^AATAMHL
A0A0A0LV53	Lysosomal beta glucosidase-like	68 kDa	NVCSNVNC^542^VVVVVSG
A0A0A0KD01	Uncharacterized protein	91 kDa	HLNAAASC^154^QIQFVCK KELDEAIC^328^WAKVSET NLEDRLAC^546^KDNSSPL
A0A0A0KTK6	Aminotran_1_2 domain-containing protein	52 kDa	KVPDVLYC^417^LKLLEAT
A0A0A0KW15	Uncharacterized protein	51 kDa	EIKEGCGC^460^KG
A0A0A0L0K6	Uncharacterized protein	50 kDa	DGVYGTTC^103^PIPPGKN
A0A0A0L0Q4	Ribos_L4_asso_C domain-containing protein	44 kDa	QGAFGNMC^100^RGGRMFA
A0A0A0L5U9	Acyl-CoA dehydrogenase family member 10	91 kDa	STVGNQMC^262^DVAYFCL NLEYGHLC^511^EIMGRSI SDATNIEC^579^SITREGD SGAMDPRC^605^KILIVMG
A0A0A0LI90	Aldedh domain-containing protein	53 kDa	HKAPIAEC^98^LVKEIAK
A0A0A0LRM4	11-beta-hydroxysteroid dehydrogenase 1B-like	38 kDa	PVETADEC^267^AKGVVRG
A0A0A0LUA8	Aldedh domain-containing protein	59 kDa	KVGPALAC^232^GNTVVLK GKSPFIVC^325^EDADVDK
A0A0A0K8W3	Uncharacterized protein	109 kDa	MKNC^4^SNALSAN KLLRNYRC^701^HPDILHL
A0A0A0KNB1	OMPdecase domain-containing protein	52 kDa	STSYDLVC^73^GVPYTAL EKIGPEIC^274^LLKTHVD
A0A0A0L1I8	DNA mismatch repair protein MLH3 isoform	136 kDa	AYVLNLEC^311^PVSFYDL KKSRMQSC^394^QASLIDS RVLNSKAC^1128^RGAIMFG
A0A0A0LAP3	Uncharacterized protein	60 kDa	MVTHC^5^INLHLHR
A0A0A0LL68	E2F_TDP domain-containing protein	47 kDa	ALALPPQC^47^CLQYHRP ACFSERQC^318^RMIIKST
A0A0A0LPD2	B5 domain-containing protein	66 kDa	ANRYDLLC^76^LEGLAQA TKNVFIEC^256^TATDLTK
A0A0A0LRD9	Programmed cell death protein 4	78 kDa	DTFEACRC^309^IRQLGVT VVSEACQC^606^IRDLGMP
A0A0A0LSH7	DEAD-box ATP-dependent RNA helicase 56	48 kDa	KDLLKNEC^166^PHIVVGT
A0A0A0LYN5	Asparagine--tRNA ligase, cytoplasmic 1	64 kDa	LQVETYAC^324^ALSSVYT DLQDDMNC^368^AEAYVRF
A0A0A0LYR4	Arginine--tRNA ligase, cytoplasmic isoform	66 kDa	AEVVEEAC^526^TNLLPNV
A0A0A0KMJ3	Uncharacterized protein	111 kDa	MARLVLPC^8^KSVGLAR QASRKLIC^80^SVATEPL DIMAKYTC^241^RIEADKS
A0A0A0KSN9	T-complex protein 1 subunit zeta 1	59 kDa	MERLVLAC^331^GGEAVNS NVKNPHSC^375^TILIKGP
A0A0A0L246	Uncharacterized protein	57 kDa	LEDTLVAC^63^LDRIFKT RSRAMVIC^278^GRLLSKE FSLVDESC^295^LRNLISA LLSSFPTC^345^VKHVIYA
A0A0A0L3I1	Peptidase_S9 domain-containing protein	81 kDa	MSPC^4^ALLRLFR VKEGDEPC^132^DITPKEF NFVDKFSC^651^PIILFQG
A0A0A0L6P6	HATPase_c domain-containing protein	80 kDa	KKSFENLC^548^KTIKDIL DRIVDSPC^573^CLVTGEY RIVDSPC^574^CLVTGEYG
A0A0A0LFM9	T-complex protein 1 subunit theta	58 kDa	KYAADAVC^516^TVLRVDQ
A0A0A0LIF5	Chaperonin CPN60-2, mitochondrial	61 kDa	VAGDGTTC^122^ATILTRA TNQKNQKC^244^ELEDPLI
A0A0A0LXZ3	UVR domain-containing protein	102 kDa	LARGELQC^404^IGATTLD
A0A0A0M2C3	RuBisCO large subunit-binding protein subunit beta, chloroplastic	64 kDa	MAVEYENC^280^KLLLVDK KTFLMSDC^584^VVVEIKE
A0A0A0KSV2	Bifunctional aspartokinase/homoserine dehydrogenase 1, chloroplastic	101 kDa	QVAVIPNC^490^SILAAVG
A0A0A0KWR4	Probable serine protease EDA2 isoform	55 kDa	MDLWLSEC^480^QSTTGRN
A0A0A0LCI7	5-methyltetrahydropteroyltriglutamate--Homocysteine methyltransferase-like	84 kDa	HLVVSTSC^328^SLLHTAV
A0A0A0LK02	SET domain-containing protein	57 kDa	RANEELIC^413^QVVRNAC
A0A0A0LZR2	5-methyltetrahydropteroyltriglutamate--homocysteine methyltransferase	91 kDa	KIVVSTSC^390^SLLHTAV
A0A0A0M063	Glyco_transf_20 domain-containing protein	97 kDa	LVKELSEC^861^SVSNLS
A0A0A0KK36	Probable polygalacturonase	48 kDa	WNIHPVYC^208^RNVVVRY
A0A0A0LHX3	Peroxisomal fatty acid beta-oxidation multifunctional protein AIM1 isoform	71 kDa	NTPQQLAC^176^IDVIEDG KVPLCIPC^201^EDKVFRE
A0A0A0LL68	legumin J	47 kDa	ALALPPQC^47^CLQYHRP ACFSERQC^318^RMIIKST
A0A0A0LNN6	Uncharacterized protein	55 kDa	NGFEETVC^313^TLRLKHS
A0A0A0L6K0	Uncharacterized protein	37 kDa	GFVFPKKC^75^NEVVIKL PEYVQKSC^147^SLNQEET AGEEGLEC^293^ISMIVAT
A0A0A0L7C4	Acetyl-coenzyme A synthetase, chloroplastic/glyoxysomal isoform	89 kDa	NLIVTSSC^10^NAVRPFP SSTTTSSC^75^LLRPPFA LAQRIIDC^329^KPKIVIT LVSHPQC^699^AEAAVVG
A0A0A0LBK4	3-ketoacyl-CoA thiolase 2, peroxisomal	47 kDa	LGTTGARC^401^VATLLSE
A0A0A0LT72	NAB domain-containing protein	40 kDa	RTSSSPSC^20^DTFSSNR KAGEMARC^248^MLKLRDD
A0A0A0LU46	Probable aspartyl aminopeptidase	56 kDa	AATNDAKC^36^KNNAVVT VVRNDMSC^449^GSTIGPI
A0A0A0LXJ8	4-hydroxy-3-methylbut-2-en-1-yl Diphosphate synthase (ferredoxin), chloroplastic	82 kDa	VALRVAEC^181^FDKIRVN
A0A0A0KGD1	Elongation factor 2-like	84 kDa	ETVEDVPC^355^GNTVAMV
A0A0A0L9F9	WD_REPEATS_REGION domain-containing protein	120 kDaa	MAC^3^IKGVNRS
A0A0A0KTQ0	PKS_ER domain-containing protein	40 kDa	PSQLNSYC^16^HFISSKL
A0A0A0KN12	Oxalate--CoA ligase-like	55 kDa	KLRFIRSC^291^SASLAPS
A0A0A0LXU2	4-coumarate--CoA ligase-like 7	59 kDa	IHSPKILC^165^FNDLVNM GRELMEEC^326^ANNIPSA
A0A0A0KEW1	Agglutinin domain-containing protein	53 kDa	ENESSWPC^93^TLFNFIP LLATKAKC^419^DIPFSYT
A0A0A0KHT0	F-box domain-containing protein	45 kDa	RLLLLRRC^66^YSTATKK
A0A0A0KLY1	ANK_REP_REGION domain-containing protein	56 kDa	MC^2^SGSKNKV KVDVNRAC^109^GSDLTTA
A0A0A0KT59	Uncharacterized protein	89 kDa	PCGLSLSC^66^SLSLSLS DKAVESLC^320^RIGSQMR AGKVTKFC^517^RILSPEL AIQHILPC^532^VKELSSD
A0A0A0KZ23	PCI domain-containing protein	37 kDa	TRNYSEKC^105^INNIMDF
A0A0A0LQN5	Minotran_1_2 domain-containing protein	52 kDa	PGNPTGQC^226^LSEANLR
A0A0A0LBA6	Starch branching enzyme I	99 kDa	FPAVPPLC^17^KRSDSTF
A0A0A0KM90	Uroporphyrinogen decarboxylase	43 kDa	MSC^3^IHNSPLP IHNSPLPC^11^FSASSSS
A0A0A0K6R4	V-type proton ATPase catalytic subunit A	68 kDa	AIPGAFGC^256^GKTVISQ
